# Feature-dependent intrinsic functional connectivity across cortical depths in the human auditory cortex

**DOI:** 10.1038/s41598-018-31292-x

**Published:** 2018-09-05

**Authors:** Pu-Yeh Wu, Ying-Hua Chu, Jo-Fu Lotus Lin, Wen-Jui Kuo, Fa-Hsuan Lin

**Affiliations:** 1 Institute of Biomedical Engineering, National Taiwan University, Taipei, 106 Taiwan; 2 Institute of Neuroscience, National Yang-Ming University, Taipei, 112 Taiwan; 3 Department of Neuroscience and Biomedical Engineering, Aalto University, Espoo, 02150 Finland

## Abstract

Frequency preference and spectral tuning are two cardinal features of information processing in the auditory cortex. However, sounds should not only be processed in separate frequency bands because information needs to be integrated to be meaningful. One way to better understand the integration of acoustic information is to examine the functional connectivity across cortical depths, as neurons are already connected differently across laminar layers. Using a tailored receiver array and surface-based cortical depth analysis, we revealed the frequency–preference as well as tuning–width dependent intrinsic functional connectivity (iFC) across cortical depths in the human auditory cortex using functional magnetic resonance imaging (fMRI). We demonstrated feature-dependent iFC in both core and noncore regions at all cortical depths. The selectivity of frequency–preference dependent iFC was higher at deeper depths than at intermediate and superficial depths in the core region. Both the selectivity of frequency–preference and tuning–width dependent iFC were stronger in the core than in the noncore region at deep cortical depths. Taken together, our findings provide evidence for a cortical depth-specific feature-dependent functional connectivity in the human auditory cortex.

## Introduction

At early stages of auditory processing, acoustic stimuli are decomposed into components at separate frequency bands^1^ and transmitted from the periphery to the cortex in spatially segregated channels^2^. This processing is supported by a topographic organization at the auditory cortical surface, where neural ensembles with similar frequency preference^3–5^ or spectral tuning^6–8^ are spatially clustered. However, generating meaningful auditory objects requires more than separating the input signals into different frequency bands, as the information needs to be somehow integrated. Previous studies have shown that integrating information may be facilitated by feature-dependent anatomical and functional connections, where neurons with similar functional properties are connected to each other across cortical locations. For example, anatomical connections between regions with similar frequency preference^9^ and tuning width^10^ were found by immunohistochemistry staining and retrograde tracing in the cat auditory cortex, respectively. Additionally, electrophysiological animal studies have reported more coherent activity between neurons with similar frequency preference in the auditory cortex of mice^11^ and monkeys^12^. One recent functional magnetic resonance imaging (fMRI) study demonstrated that the selectivity of frequency-dependent intrinsic functional connectivity (iFC) is higher in the core than in the noncore region of the human auditory cortex^13^, which is closely related to the hierarchical organization of the auditory cortex for the processing of spectrally complex stimuli. In the auditory cortex, inter- and intra-laminar anatomical connections have been found between columnarly organized neurons in the direction perpendicular to the cortical surface^14–16^. Concerting with the diverse anatomical connections across laminar layers, electrophysiological recordings showed laminar layer-specific connectivity patterns in the cat auditory cortex. Specifically, functionally connected neurons have more similar spectrotemporal receptive fields (STRF)^17^, frequency preference^18^, firing rate, and best temporal modulation frequency^19^ at supragranular layers than at infragranular layers, while the higher functional similarity of paired versus unpaired neurons is most prominent in infragranular layers. However, it remains unclear how auditory information is integrated across cortical depths in humans.

Recent advances in fMRI acquisition and analysis methods allow for the characterization of hemodynamic responses across cortical depths in the human brain, thus providing improvements for the study of functional specificity^20^. Specifically, examining blood-oxygen-level dependent (BOLD) signal with a small voxel in the range of 1 µL can improve functional specificity by alleviating the vascular bias caused by draining veins coursing along the pial surface^20,21^, by reducing partial volume effects^22,23^, and by suppressing physiological noise^24^. Accordingly, a number of high spatial-resolution fMRI studies have successfully detected cortical depth-specific functional activity in the human visual cortex^25–32^. Similar experimental protocols have also been used to reveal how functions of the human auditory cortex differ across cortical depths^21,33,34^. While the frequency preference, tuning width, and top-down attentional modulation effects have been examined across cortical depths, how the iFC in the human auditory cortex varies across cortical depths is unknown.

In this study, we characterized the feature-dependent iFC across cortical depths in the human auditory cortex. We specifically examined how iFC depends on the difference in frequency preference and tuning width within different cortical depths, respectively, in both core and noncore regions of the human auditory cortex. Frequency preference and tuning width were chosen as the independent variables because these two acoustic dimensions are represented by spatially segregated neuronal ensembles^35^, and this representation has been suggested to facilitate simultaneous processing of local and global spectral information^10^. In humans, iFC has been found to be more selective in the core than in the noncore region^13^, yet how feature-dependent iFC change across cortical depths, particularly how this change varies between core and noncore regions, remains elusive. Based on previous invasive animal studies showing the laminar layer-specific functional connections^17–19^, particularly the higher functional similarity difference between paired and unpaired neurons at infragranular layers of the primary auditory cortex, we hypothesize that the selectivity of feature-dependent iFC in the core region is higher at deep cortical depths. This feature can be more distinct when contrasting between core and noncore regions, as more associative operations occur and thus less selective iFC is needed in the noncore region^36,37^.

## Results

### Verification of approaches for cortical depth analysis at the auditory cortex

**Figure 1:**
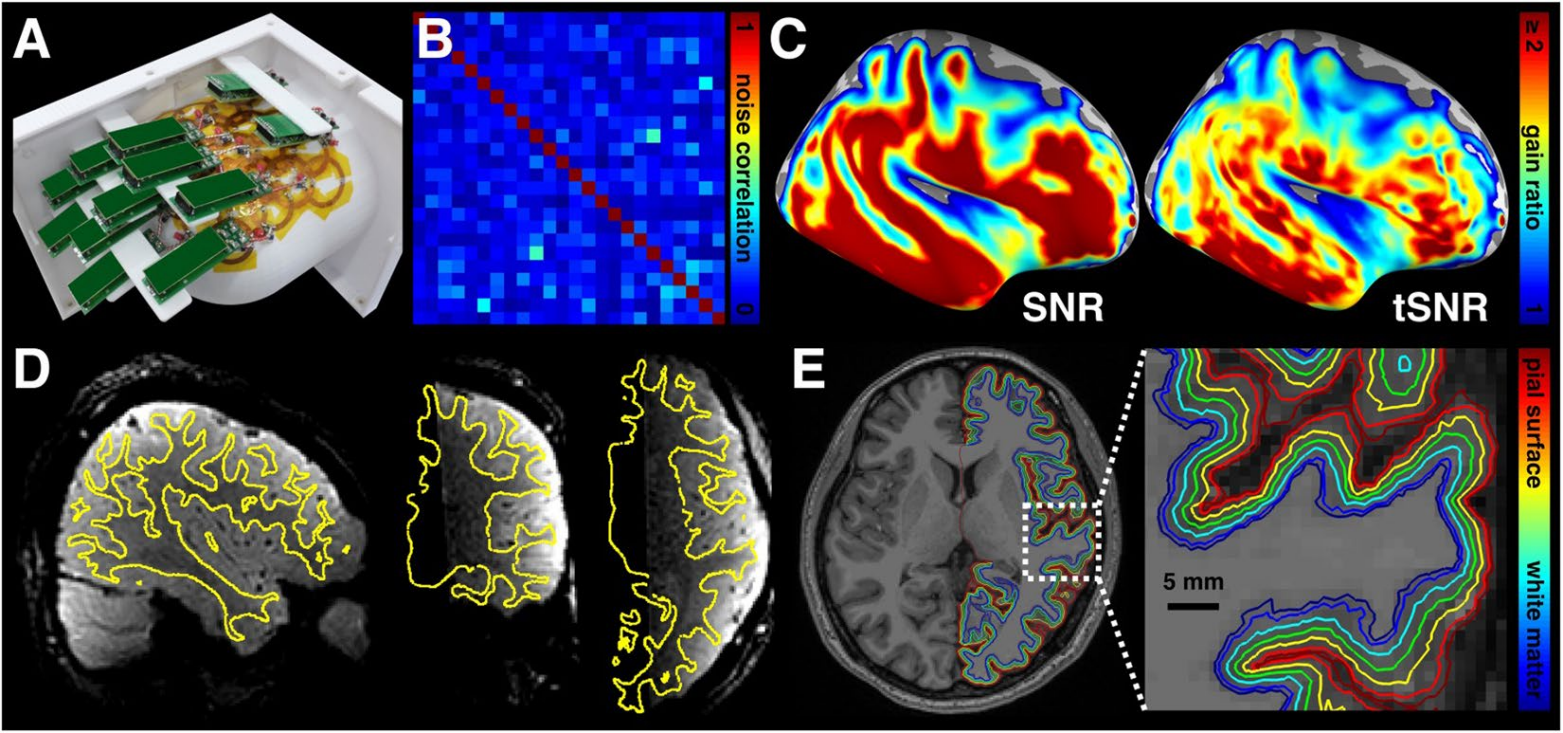
Experimental setup and surface-based cortical depth analysis. (**A**) A 24-channel temporal lobe coil array with the mechanical housing, including 24 coil elements arranged hexagonally and critically overlapped to cover the right temporal lobe. (**B**) The noise correlation matrix of the coil array. (**C**) Spatial distributions of the SNR and tSNR gain ratio between a 24-channel temporal lobe array and a 32-channel whole-head array. (**D**) Registration between functional and anatomical images. The yellow contour denotes the gray-white matter interface reconstructed from the anatomical image. (**E**) An axial slice of the anatomical image superimposed with seven reconstructed cortical surfaces ranging from the white matter to the pial surface. The blue contour represents the gray-white matter boundary. The red contour represents the gray-pial surface boundary.

**Figure 2:**
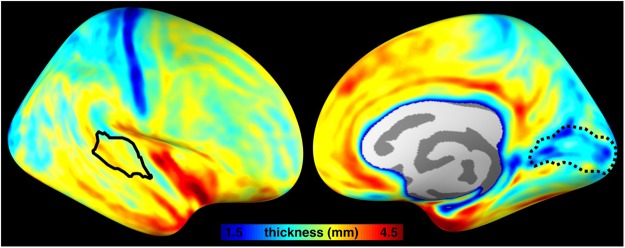
Spatial distributions of the averaged cortical thickness (*n* = 20) in the right hemisphere. The auditory cortex and the visual cortex (the “V1” region suggested by FreeSurfer) are indicated by the solid and dotted black contours, respectively.

In order to optimize the signal-to-noise ratio (SNR) for the study on the human auditory cortex across cortical depths using 3T MRI, we constructed a dedicated 24-channel temporal lobe coil array (Fig. 1A). Figure 1B shows the noise correlation matrix of the coil array between 24 coil channels. The minimum, maximum, and the average of off-diagonal entries were 0.01, 0.43, and 0.11, respectively. Figure 1C shows the SNR and temporal SNR (tSNR) gain ratio maps (with respect to a commercial 32-channel whole-head array). At least 40% improvement in most of the temporal lobe was found by visual inspection. Quantitatively, our array provided a SNR gain of 1.90 ± 0.49 and a tSNR gain of 1.69 ± 0.30 in the region of interest (ROI) at the auditory cortex (denoted by the solid contour in Fig. 2).

Cortical depth analysis included verifying accuracy of the registration between functional and anatomical images. Figure 1D shows three representative slices in orthogonal views, superimposed with the gray-white matter boundary estimated from anatomical images. Good registration between functional and anatomical images was found by visual inspection. An example of contours for different cortical depths from a representative participant is shown in Fig. 1E. The gray-white matter boundary, the gray-pial surface boundary, and five intermediate surfaces (denoted by a normalized distance from the white matter boundary; nd = 0.1, 0.3, 0.5, 0.7, 0.9), were overlaid on top of the anatomical image. We also computed the averaged cortical thickness from our participants to ensure that the 1.5 mm isotropic spatial resolution was sufficient for the cortical depth analysis (Fig. 2). Quantitatively, the cortical thickness was 3.29 ± 0.20 mm in the auditory cortex ROI, and 2.48 ± 0.16 mm in the visual cortex ROI. Note that when using the 1.5 mm isotropic spatial resolution there were more than two voxels across cortical depths in the auditory cortex, comparable to a previous study^20^, in which 1 mm isotropic resolution was used to study fMRI signals across cortical depths at the visual cortex.

### Analysis of functional properties across cortical depths

**Figure 3:**
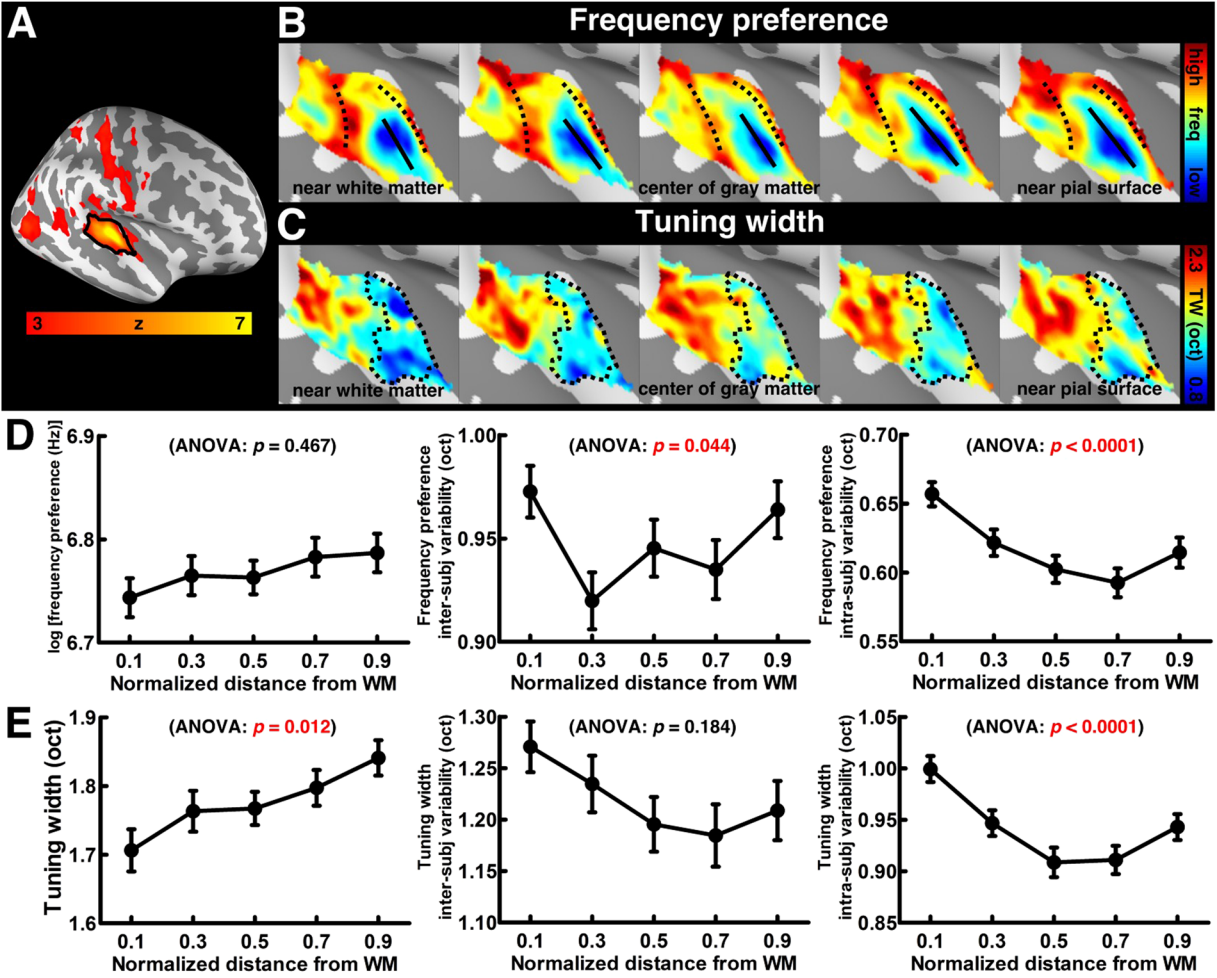
Cortical depth analysis of the frequency preference and tuning width. (**A**) Spatial distributions of the averaged z-scores on the right hemisphere, with the black contour denoting the auditory cortex ROI. (**B**) Spatial distributions of the frequency preference at five representative cortical depths of the auditory cortex, with solid and dotted lines indicating the low- and high-frequency preference bands, respectively. (**C**) Spatial distributions of the tuning width at the auditory cortex, with the dotted contour indicating the region with a narrow tuning width. (**D**) Profiles of the frequency preference and its variabilities at five cortical depths. (**E**) Profiles of the tuning width and its variabilities at five cortical depths. In panels (D,E), error bars represent the standard error of the mean (SEM) across cortical locations in the auditory cortex ROI. The significance of the difference across cortical depths was quantified by one-way ANOVA. freq = frequency, TW = tuning width, oct = octave, WM = white matter.

**Figure 4:**
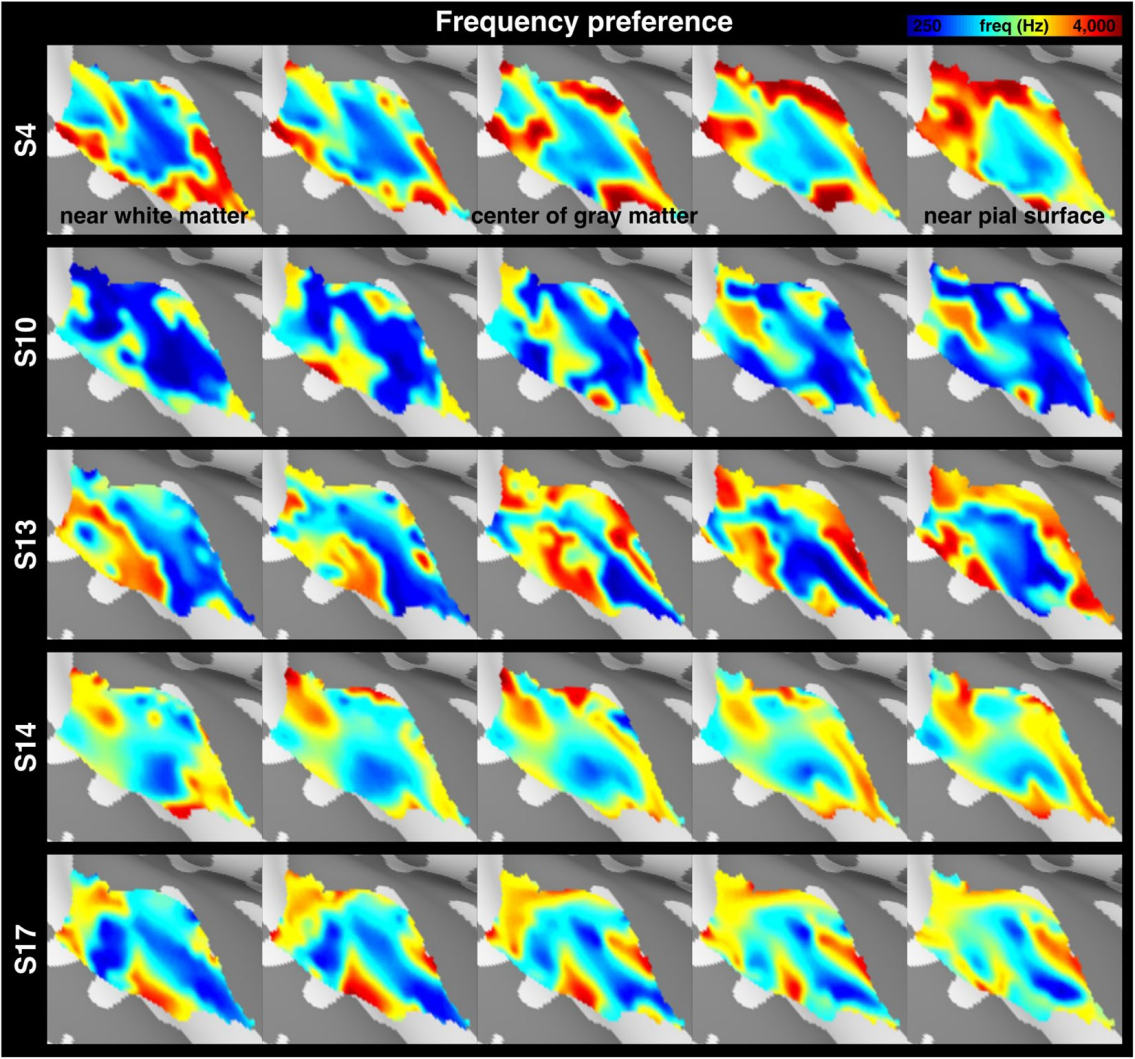
Spatial distributions of the frequency preference from five representative participants at five cortical depths of the auditory cortex.

**Figure 5:**
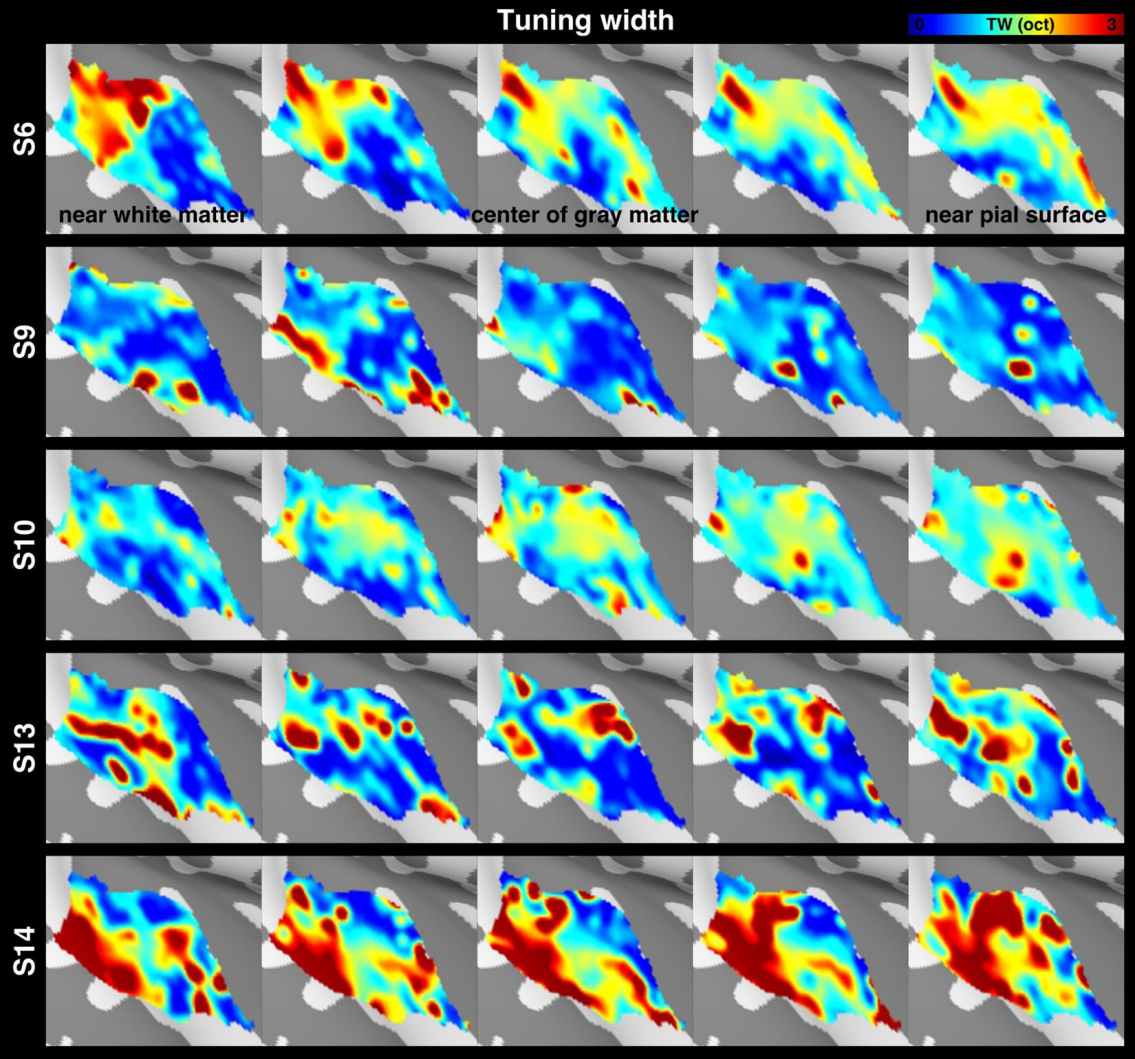
Spatial distributions of the tuning width from five representative participants at five cortical depths of the auditory cortex.

After projecting the functional time series to each participant’s five intermediate cortical surfaces, we estimated the average and the variability of functional properties across cortical depths. The chirp tone elicited robust BOLD responses in the auditory cortex. Figure 3A shows the spatial distribution of z-scores, which quantified how empirically measured fMRI time series fit to the predicted model. This map was derived from 20 participants and five cortical depths. Figure 3B shows spatial distributions of the mean frequency preference across participants (results from individual participants are shown in Fig. 4), which was converted from local fMRI response latency, at five cortical surfaces. The tonotopic representations clearly present one low (solid line) and two high (dotted line) frequency preference bands extending along the superior-to-inferior axis. These frequency-selective bands suggest a mirror-symmetric high-low-high frequency-gradient topology perpendicular to the Heschl’s gyrus. The spatial distributions of the mean tuning width across participants (Fig. 3C; results from individual participants are shown in Fig. 5) show a narrowly tuned region (dotted contour) extended along the Heschl’s gyrus, and a broader tuning area located at the Heschl’s sulcus and superior temporal gyrus. While the spatial distribution of the frequency preference map was roughly constant across cortical depths, the tuning width appeared to be different across cortical depths.

Quantitatively, the frequency preference (Fig. 3D left) was constant across cortical depths (one-way ANOVA: *F* (4,705) = 0.895, *p* = 0.467). In contrast, the tuning width (Fig. 3E left) showed significant difference across cortical depths (one-way ANOVA: *F* (4,705) = 3.243, *p* = 0.012). Specifically, the tuning widths at the deep and intermediate depths were significantly smaller than that at the superficial depth (one-tailed *t*-test: nd–nd = 0.1–0.7, *p* = 0.043; nd–nd = 0.1–0.9, *p* = 0.003; nd–nd = 0.3–0.9, *p* = 0.044; nd–nd = 0.5–0.9, *p* = 0.044). These results are consistent with previous fMRI findings^21,33,34^ and also neurophysiological animal studies showing a columnar frequency preference and laminar layer-specific tuning width organization^38^. We further analyzed the variability of frequency preference and tuning width. The inter-subject variability of frequency preference differed significantly across cortical depths (Fig. 3D middle, one-way ANOVA: *F* (4,705) = 2.463, *p* = 0.044). The inter-subject variability at the intermediate depth was significantly smaller than that at the deep and superficial depths (one-tailed *t*-test: nd–nd = 0.1–0.3, *p* = 0.017; nd–nd = 0.3–0.9, *p* = 0.042). The intra-subject variability of frequency preference also showed a significant difference across cortical depths (Fig. 3D right, one-way ANOVA: *F* (4,705) = 6.077, *p* < 0.0001). The intra-subject variabilities at the intermediate and superficial depths were significantly smaller than that at the deep depth (one-tailed *t*-test: nd–nd = 0.1–0.3, *p* = 0.006; nd–nd = 0.1–0.5, *p* < 0.0001; nd–nd = 0.1–0.7, *p* < 0.0001; nd–nd = 0.1–0.9, *p* = 0.003). These results corroborated with the previous finding^21^ that the superficial depth of the auditory cortex has a reletively large inter-subject variability due to more physiological and anatomical bias imparted by the venous vasculature towards the pial surface. However, the lowest inter- and intra-subject variability were found in the intermediate cortical depth. We refer this result to the neurophysiological organization that the specificity of frequency preference is highest in the granular layer, where thalamic projections terminate^39,40^. Inter- and intra-subject variabilities of tuning width show the same trend as those in the frequency preference variabilities, while only the intra-subject variability of tuning width shows a significant difference across cortical depths (Fig. 3E middle, one-way ANOVA: *F* (4,705) = 1.559, *p* = 0.184; Fig. 3E right, one-way ANOVA: *F* (4,705) = 7.718, *p* < 0.0001). The intra-subject variabilities at the intermediate and superficial depths were significantly smaller than that at the deep depth (one-tailed *t*-test: nd–nd = 0.1–0.3, *p* = 0.003; nd–nd = 0.1–0.5, *p* < 0.0001; nd–nd = 0.1–0.7, *p* < 0.0001; nd–nd = 0.1–0.9, *p* = 0.002).

### Structural and functional examination of core and noncore regions

**Figure 6:**
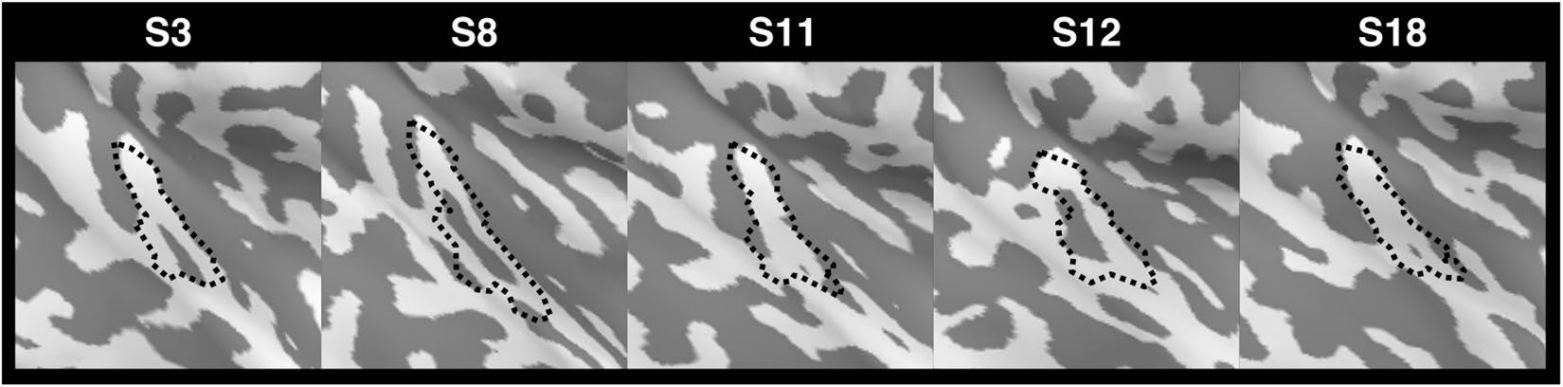
Back projection of the group-level-defined core region (dotted contour) onto the individual cortical surfaces for five representative participants.

The narrowly tuned area shown in Fig. 3C was defined as the core region of the auditory cortex for following feature-dependent iFC analysis. Considering the morphological variability of the Heschl’s gyrus across participants^41^, we back projected the group-level-defined core region onto the cortical surface of individual participants. Results from five representative participants are shown in Fig. 6, indicating a good alignment of back projected core region with individuals’ Heschl’s gyrus. To further reduce the concern of defining the core and noncore regions from group data, we calculated the frequency preference and tuning width across cortical depths separately in the core and noncore regions. The log (frequency preference) in the core region at different cortical depths were 6.61 ± 0.25 (nd = 0.1), 6.61 ± 0.23 (nd = 0.3), 6.67 ± 0.23 (nd = 0.5), 6.71 ± 0.28 (nd = 0.7), and 6.72 ± 0.27 (nd = 0.9). The log (frequency preference) in the noncore region at different cortical depths were 6.82 ± 0.17 (nd = 0.1), 6.85 ± 0.17 (nd = 0.3), 6.81 ± 0.16 (nd = 0.5), 6.82 ± 0.18 (nd = 0.7), and 6.82 ± 0.18 (nd = 0.9). Although the core region preferred significantly lower frequency than the noncore region, the differences were consistent across cortical depths (one-tailed *t*-test: *p* < 0.05). Similar results were found in the tuning width data. The tuning width in the core region at different cortical depths were 1.43 ± 0.27 (nd = 0.1), 1.49 ± 0.23 (nd = 0.3), 1.56 ± 0.22 (nd = 0.5), 1.55 ± 0.24 (nd = 0.7), 1.62 ± 0.25 (nd = 0.9). The tuning width in the noncore region at different cortical depths were 1.86 ± 0.32 (nd = 0.1), 1.92 ± 0.32 (nd = 0.3), 1.89 ± 0.26 (nd = 0.5), 1.94 ± 0.25 (nd = 0.7), 1.97 ± 0.27 (nd = 0.9). The tuning widths in the core were consistently significantly smaller than those in the noncore region at each cortical depth (one-tailed *t*-test: *p* < 0.05). Note that the narrowest tuning width was found at the deepest cortical depth in both core and noncore regions.

Likewise, the inter-subject variability of frequency preference in the core region at different cortical depths were 1.00 ± 0.18 (nd = 0.1), 0.95 ± 0.20 (nd = 0.3), 0.97 ± 0.18 (nd = 0.5), 0.97 ± 0.17 (nd = 0.7), 1.02 ± 0.15 (nd = 0.9). The inter-subject variability of frequency preference in the noncore region at different cortical depths were 0.96 ± 0.12 (nd = 0.1), 0.90 ± 0.14 (nd = 0.3), 0.93 ± 0.16 (nd = 0.5), 0.92 ± 0.17 (nd = 0.7), 0.93 ± 0.16 (nd = 0.9). Comparing between two regions, the inter-subject variability was significantly larger in core than in the noncore region only at the superficial depth (one-tailed *t*-test: nd = 0.9, *p* < 0.05). Since we used the same ROI across cortical depths, if the ROI mixed data between core and noncore regions, we would expect to observe insignificant difference across all cortical depths. The observation of significant inter-subject variability difference at the superficial cortical depth ruled out such speculation. The inter-subject variability of tuning width in the core region at different cortical depths were 1.14 ± 0.32 (nd = 0.1), 1.11 ± 0.32 (nd = 0.3), 1.08 ± 0.32 (nd = 0.5), 1.00 ± 0.33 (nd = 0.7), 1.07 ± 0.35 (nd = 0.9). The inter-subject variability of tuning width in the noncore region at different cortical depths were 1.35 ± 0.25 (nd = 0.1), 1.31 ± 0.31 (nd = 0.3), 1.26 ± 0.30 (nd = 0.5), 1.29 ± 0.34 (nd = 0.7), 1.29 ± 0.31 (nd = 0.9). We found significantly smaller inter-subject variability in the core than that in the noncore region (one-tailed *t*-test: *p* < 0.05). However, the differences were also consistent across cortical depths. Such a significant difference between our group-level-defined core and noncore regions suggested that the potential bias due to different core and noncore region boundaries across participants was minimal.

### Analysis of feature-dependent intrinsic functional connectivity across cortical depths

**Figure 7:**
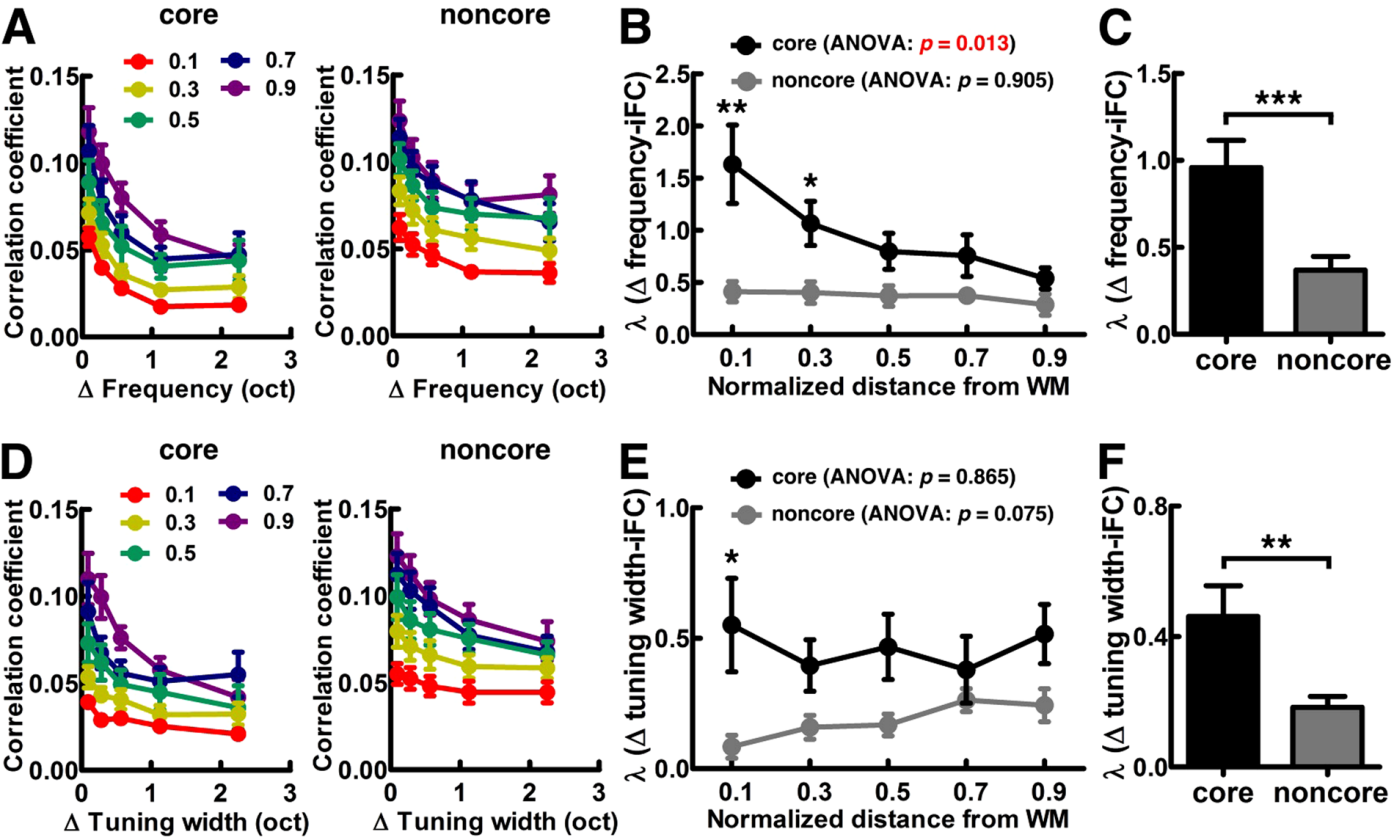
Feature-dependent iFC analysis in core and noncore regions of the auditory cortex. (**A**) Functional connectivity measured by correlation coefficients as a function of Δ frequency in core (left) and noncore (right) regions of the auditory cortex. (**B**) Profiles of λs in fitting the Δ frequency-iFC data in both core and noncore regions at five cortical depths. (**C**) Profiles of λs in fitting the Δ frequency-iFC data obtained by averaging across cortical depths. (**D**) Functional connectivity measured by correlation coefficients as a function of Δ tuning width in core (left) and noncore (right) regions of the auditory cortex. (**E**) Profiles of λs in fitting the Δ tuning width-iFC data in both core and noncore regions at five cortical depths. (**F**) Profiles of λs in fitting the Δ tuning width-iFC data obtained by averaging across cortical depths. In all panels, error bars represent SEM across participants. The significance of the difference across cortical depths was quantified by one-way ANOVA, and the significance of the difference between λs in core and noncore regions was quantified by one-tailed *t*-test (* for *p* < 0.05, ** for *p* < 0.01, and *** for *p* < 0.001).

Figure 7A shows the iFC as a function of difference in frequency preference (Δ frequency) within core and noncore regions of the auditory cortex. Consistent with a previous study^13^, we found that at each of the five cortical depths, the iFC gradually decreased as Δ frequency increased in both core and noncore regions (Page’s trend test: *p* < 0.0001). We then investigated whether the degree of selectivity of frequency–preference dependent iFC differs across cortical depths and regions. The selectivity of frequency–preference dependent iFC was quantified by a constant of an exponential decay function fitted to the Δ frequency-iFC data. Figure 7B shows the fitted λ in core and noncore regions of the auditory cortex at different cortical depths. Quantitatively, while the selectivity of frequency–preference dependent iFC was constant across depths in the noncore region (one-way ANOVA: *F* (4,95) = 0.257, *p* = 0.905), it varied significantly across cortical depths in the core region (one-way ANOVA: *F* (4,95) = 3.340, *p* = 0.013). In particular, the λ at the deep depth was found significantly larger than that at the intermediate and superficial depths (one-tailed *t*-test: nd–nd = 0.1–0.5, *p* = 0.044; nd–nd = 0.1–0.7, *p* = 0.044; nd–nd = 0.1–0.9, *p* = 0.027). The λ at the relatively deep intermediate depth was also found significantly larger than that at the superficial depth (one-tailed *t*-test: nd–nd = 0.3–0.9, *p* = 0.044). Comparing between core and noncore regions, we found that the λs of frequency–preference dependent iFC in the core were significantly higher than that in the noncore region at the deep (one-tailed *t*-test: nd = 0.1, *p* = 0.008) and relatively deep intermediate (one-tailed *t*-test: nd = 0.3, *p* = 0.020) depths. After averaging across cortical depths, we found that the selectivity of frequency–preference dependent iFC in the core region was significantly higher than that in the noncore region (Fig. 7C, one-tailed *t*-test: *p* = 0.001). This result was consistent with a previous study^13^.

**Figure 8:**
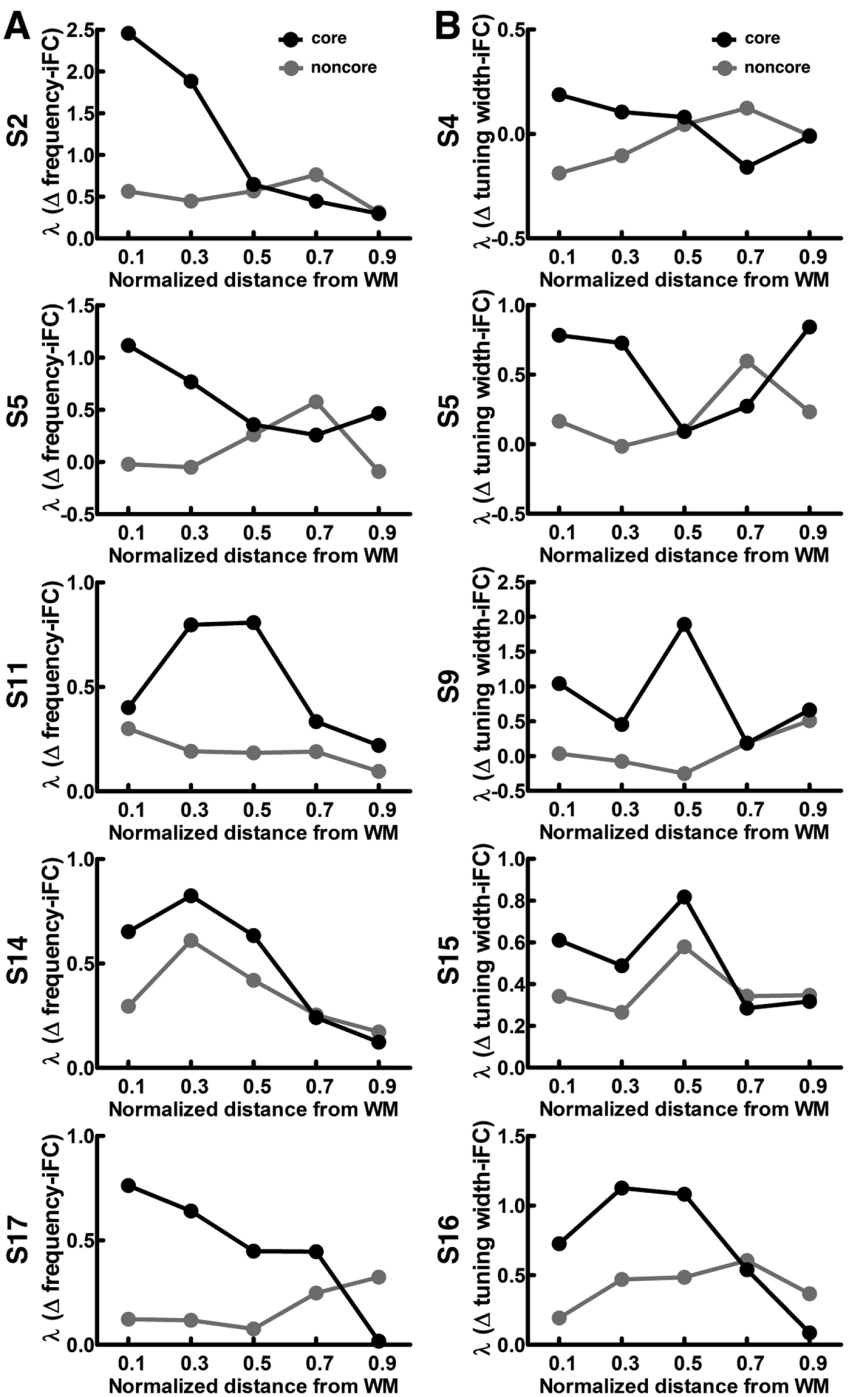
Selectivity of feature-dependent iFC in individual participants. (**A**) Profiles of λs in fitting the Δ frequency-iFC data from five representative participants are plotted in the same format as Fig. 7B. (**B**) Profiles of λs in fitting the Δ tuning width-iFC data from five representative participants.

We also examined the iFC as a function of difference in tuning width (Δ tuning width). We found that the iFC gradually decreased as Δ tuning width increased in both core and noncore regions at each of the five different depths (Fig. 7D, Page’s trend test: *p* < 0.001). Figure 7E shows that the selectivity of tuning–width dependent iFC was constant across cortical depths in both core (one-way ANOVA: *F* (4,95) = 0.319, *p* = 0.865) and noncore (one-way ANOVA: *F* (4,95) = 2.202, *p* = 0.075) regions. The selectivity of tuning–width dependent iFC across cortical depths substantiates the frequency–preference dependent iFC results: selectivity of tuning–width dependent iFC in the core region was significantly higher than that in the noncore region at the deep (one-tailed *t*-test: nd = 0.1, *p* = 0.039) cortical depth. After averaging across cortical depths, we found that the selectivity of tuning–width dependent iFC in the core region was significantly higher than that in the noncore region (Fig. 7F, one-tailed *t*-test: *p* = 0.004). The selectivity of feature-dependent iFC from five representative participants is shown in Fig. 8, demonstrating the consistency of the results across individual participants.

**Figure 9:**
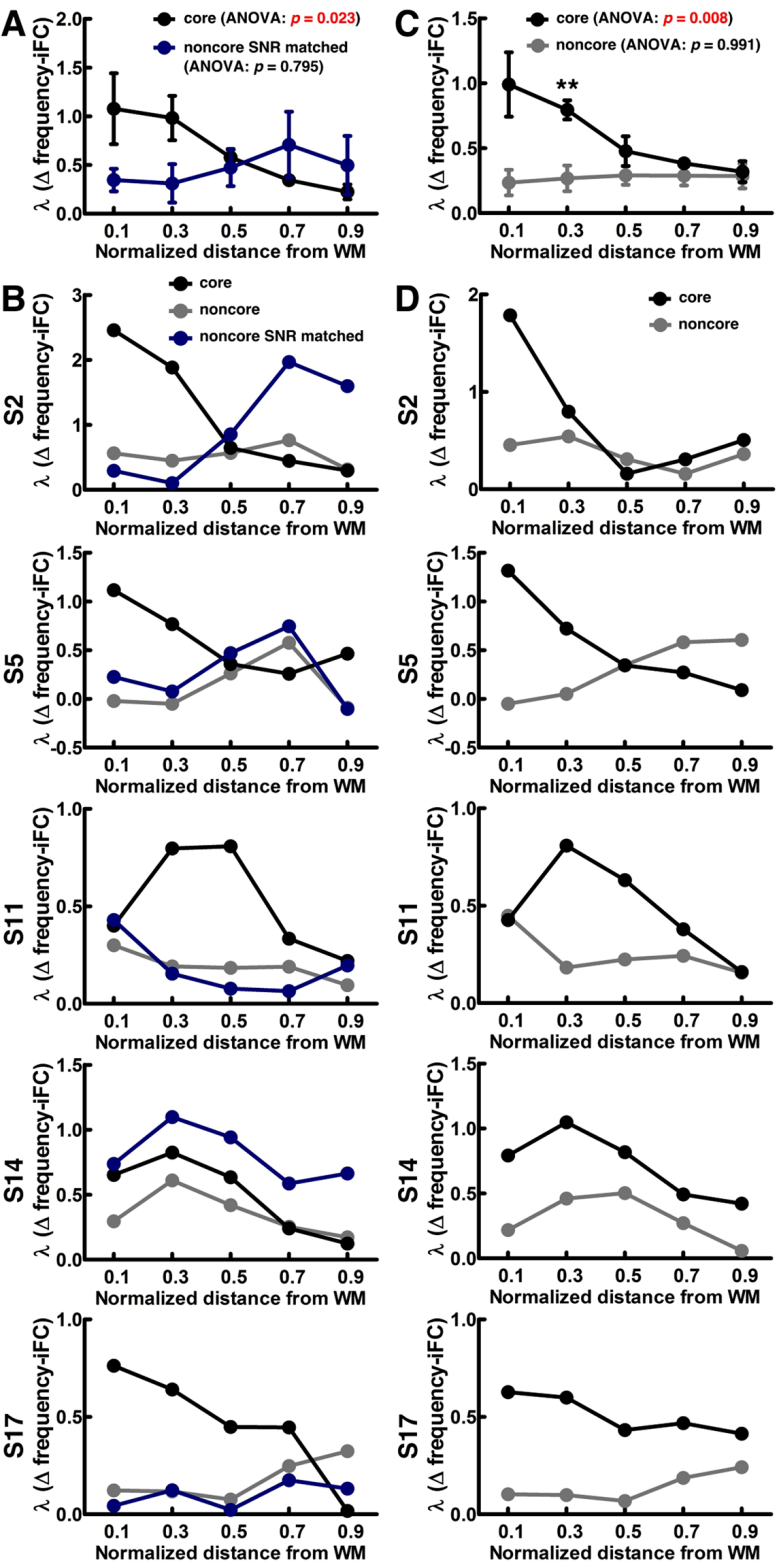
Testing potential confounding factors in feature-dependent iFC analysis. (**A**) Profiles of λs in fitting the Δ frequency-iFC data in core and noncore SNR matched ROIs. (**B**) Profiles of λs in fitting the Δ frequency-iFC data in core, noncore, and noncore SNR matched ROIs from five representative participants. (**C**) Profiles of λs using group data in fitting the Δ frequency-iFC data with the time series pre-processed by bandpass-filtering (0.01 Hz to 0.1 Hz), regression with motion translation and rotation, and regression with white matter signal. (**D**) Profiles of λs from five representative participants in fitting the Δ frequency-iFC data with the time series pre-processed by bandpass-filtering (0.01 Hz to 0.1 Hz), regression with motion translation and rotation, and regression with white matter signal. In panels (A,C), error bars represent SEM across participants. The significance of the difference across cortical depths was quantified by one-way ANOVA, and the significance of the difference between λs in core and noncore regions was quantified by one-tailed *t*-test (* for *p* < 0.05, ** for *p* < 0.01, and *** for *p* < 0.001).

Due to the fact that we found our coil array provided a significant smaller (one-tailed *t*-test: *p* < 0.05) SNR in the core (163.37 ± 59.64) than the noncore (238.40 ± 76.26) region, we further tested if the SNR difference influences the feature-dependent iFC results. We selected a noncore SNR matched ROI (in the medial portion of the original noncore ROI) with a comparable SNR (181.12 ± 49.22) to the core region while maintaining the voxel number as in the core region. Then we did the frequency–preference dependent iFC analysis on five representative participants. Both group (Fig. 9A) and individual (Fig. 9B) λ profiles of frequency–preference dependent iFC show very similar results comparing to the Figs 7B and 8A. These results indicate that the SNR difference between core and noncore regions did not influence the feature-dependent iFC results. In addition, to study whether other confounding factors such as motion and physiological noise affect the feature-dependent iFC results, here we further pre-processed our time-series data by using a 6th-order Butterworth bandpass-filter between 0.01 and 0.1 Hz. We also included six motion regressors and a white matter regressor (the average time series of a 1 cm^3^ cube in the white matter) in the GLM to remove potential confounding factors. Then we did the frequency–preference dependent iFC analysis on five representative participants. Both group (Fig. 9C) and individual (Fig. 9D) λ profiles of frequency–preference dependent iFC show very similar results comparing to the Figs 7B and 8A. These results indicate that the motion and physiological noise did not influence the feature-dependent iFC results.

## Discussion

This study delineates cortical depth-specific functional connectivity in the human auditory cortex. Our analysis of iFC found that residual activities between brain locations with more similar frequency preference (Fig. 7A) and tuning width (Fig. 7D) are more correlated. While this feature-dependent iFC exists in both core and noncore regions at all cortical depths, the degree of selectivity of feature-dependent iFC shows a significant difference across cortical depths in the core region (Fig. 7B). Specifically, the selectivity of frequency–preference dependent iFC is stronger at deep than that at the intermediate and superficial depths. Comparing the selectivity of frequency–preference and tuning–width dependent iFC between the core and noncore regions, we observed that both the selectivity of frequency–preference and tuning–width dependent iFC were signifcantly stronger in the core than that in the noncore region at deep cortical depths (Fig. 7B,E). These results were not explained by SNR difference between the core and noncore regions (Fig. 9A,B), or by other confounding factors, such as motion and physiological noise (Fig. 9C,D). Taken together, we found a stronger selectivity of feature-dependent iFC in the core than in the noncore region as we moved from superficial to deep cortical depths. Previously, an invasive animal study revealed that the difference in functional similarity between functionally paired and unpaired neurons is higher at infragranular layers in the cat primary auditory cortex^19^. Our results echoed this finding by demonstrating the relationship between functional similarity and iFC at deep cortical depths. The top-down feedback pathway has been reported to target the infragranular and supragranular layers^42,43^. A recent study also showed that a feedback activity induced by illusory figures led to a selective activation in the deep human primary visual cortex^30^. Thus, our findings suggested that the top-down modulation in the human auditory cortex is underpinned by an architecture of stronger selectivity of feature-dependent iFC in the deep cortical depth. We speculate that such an architecture is helpful in selectively activating or deactivating specific neuronal ensembles during sensory processing.

Compared to other cortical depth-specific fMRI studies on the human auditory cortex using 1 mm or higher isotropic resolution^21,33,34^, we used 1.5 mm isotropic resolution, which may correspond to only two independent voxels across cortical depths. A larger image voxel has a stronger signal at the cost of functional specificity. We considered the 1.5 mm resolution sufficient for supporting the results reported here, because (1) surface-based cortical depth analysis takes advantage of the highly folded and curved geometry of the human cortex, the spatial extension of which results in variabilities of the number of EPI voxels intersecting the cortical surfaces across depths^20^, (2) accordingly, this fMRI study in the human visual cortex across cortical depths suggest that image resolution was about ½ the cortical thickness, (3) the human auditory cortex is about 50% thicker (~2.8 mm) than the visual cortex (~1.8 mm)^44–46^, and (4) cortical depth-specific functional activity was found in the human hippocampus and entorhinal cortex by using data acquired at a 0.8 mm isotropic resolution but spatially smoothed with a 1.5 mm Gaussian kernal^47^. We also used a tailored 24-channel receiver coil array to acquire fMRI data in order to compensate for the relative lower sensitivity at 3T than at 7T (with a whole-head array). Of importance to note is that our results are highly consistent with those from previous studies done at 7T with 1mm isotropic or higher resolution by showing that, first, the frequency preference was constant across cortical depths^21,33,34^ (Fig. 3D). Second, the tuning width was significantly different across cortical depths: deep and intermediate depths had significantly narrower tuning widths than superficial depths^33,34^ (Fig. 3E). Third, the inter-subject variabilities of the frequency preference was found relatively larger at the superficial depth^20,21^ (Fig. 3D). In sum, these findings suggest that our imaging protocol included sufficient sensitivity and specificity for revealing cortical depth-specific functional characteristics.

In this study, we used relatively simple and artificial stimuli to elicit brain responses. These stimuli facilitated the analysis of frequency–preference and tuning–width dependent iFC. However, cortical depth-specific functional connections may also involve other complex acoustic characteristics, such as STRF structure^17^ and preferred spectral and temporal modulation frequency^19^. Further studies in the functional connectivity associated with these acoustic features may help elucidate how the brain encodes and integrates information when receiving complex and naturalistic auditory inputs.

In this study, the iFC was derived from the residual fMRI. Note that previously it was shown that the iFC from residual fMRI signals was similar to the iFC from the resting state^13^. However, it may be also interesting to explore the cortical depth-specific functional connectivity during behavioral task engagement or under different cognitive conditions. For example, one study showed that the functional connectivity networks involved in early visual perception are modified by dinstinct task requirements^48^. In addition, electrophysiological animal studies revealed that neuronal synchronization can be modulated by attentional state^49,50^ and adaptation^51^ in a laminar layer-specific manner. How these modulations of functional connectivity vary across human cortical depths should be addressed in future studies.

As suggested by recent studies^34,52^, cortical depth fMRI data acquired by a gradient echo EPI sequence may be biased by vasculature and vascular reactivity. Thus, we cannot attribute the iFC characteristics found here solely to neuronal responses. This bias can nevertheless be reduced by a tailored pulse sequence (at the cost of reduced sensitivity)^52,53^, cortical depth-specific vasculature and vascular reactivity mapping, or combining fMRI and invasive electrophysiological measurements, such as cortical depth-specific electrode recording, to further clarify the physiological origin of the iFC characteristics found here.

## Methods

### Participants

Twenty healthy subjects (age: 26.6 ± 6.3; 9 males) participated in this study. All participants had no history of hearing disorders or neurological disease. All methods were carried out in accordance with guidelines and regulations of National Taiwan University Hospital. All experimental protocols were approved by the Institutional Review Board of National Taiwan University Hospital. Informed consent was obtained from all participants.

### Auditory stimulation

The auditory stimuli consisted of a 20 sec logarithmic tone chirp with a frequency span from 250 Hz to 4,000 Hz, followed by a 10 sec silent period. During each imaging run, the 30 sec stimuli was repeated 15 times, forming a presentation frequency of 0.033 Hz. Four runs of auditory stimulus presented to the participant had two rising chirp cycles (beginning at 250 Hz and ending at 4,000 Hz) and two falling chirp cycles (beginning at 4,000 Hz and ending at 250 Hz). The stimuli were delivered binaurally using the Psychophysics Toolbox^54^ by MATLAB (MathWorks, Natick, MA, USA) via a MR-compatible insert earphone (Model S14, Sensimetrics, MA, USA). Acoustic scanner noise was further attenuated by earmuffs placed over the ears after the earphone insertion. The stimuli intensity was kept constant across frequencies and was set individually at levels between 75 and 85 dB SPL so that participants could hear the entire chirp clearly on top of the scanner noise.

### Coil array construction and MRI acquisition

All data were acquired on a 3T MRI system (Skyra, Siemens Healthcare, Erlangen, Germany). We developed a dedicated 24-channel surface coil array with 50 mm diameter loops to optimize the SNR for fMRI at the temporal lobe. Details of the RF coil circuitry have been described in Chu *et al*.^55^. A noise correlation matrix was estimated using data acquired from a zero-degree flip angle pulse sequence (FOV: 256 × 256 mm^2^; TR = 100 ms; TE = 30 ms; BW = 2520 Hz/pixel; matrix = 64 × 64; slice thickness = 256 mm; 1 axial slice). SNR and tSNR were calculated using data acquired from a 1.5 mm isotropic resolution gradient-echo EPI protocol with GRAPPA acceleration^56^ (FOV: 192 × 192 mm^2^; TR = 2500 ms; TE = 28 ms; FA = 90°; BW = 1260 Hz/pixel; 38 sagittal slices; R = 3). SNR was calculated by first using the GRAPPA approach to reconstruct fully sampled *k*-space data for each coil element in the array. Then these images were combined using the noise-covariance weighted root sum-of-squares reconstruction^57^. tSNR was calculated by taking the ratio between the mean and the standard deviation of the time series. For functional image acquisition, we applied the same 1.5 mm isotropic resolution EPI, and the temporal lobe array for reception. Due to the coil loop arrangement, the FOV was restricted to only the right hemisphere. A gradient-echo field-mapping scan (FOV: 192 x 192 mm^2^; TR = 1000 ms; TE (short/long) = 10/12.46 ms; FA = 90°; BW = 260 Hz/pixel; 19 sagittal slice) with the same FOV as EPI was also performed to collect data for distortion correction. Structural images for each participant were acquired using a whole-head 32-channel coil array and a 1 mm isotropic resolution *T*_1_-weighted MPRAGE sequence (FOV: 256 × 256 mm^2^; TR = 2530 ms; TE = 3.3 ms; TI = 1100 ms; FA = 7°; BW = 200 Hz/pixel; 192 sagittal slice).

### Cortical surface reconstruction

The triangulated mesh surfaces of gray-white matter boundary, gray-pial surface boundary, and nine cortical depths with equally spaced cortical thickness were automatically reconstructed from the MPRAGE images using FreeSurfer^20,58,59^. The cortical thickness maps were derived from the gray-white matter and gray-pial surface boundaries^44^. Then we took surface 2, 4, 6, 8, and 10 out of these 11 surfaces (normalized distance from the white matter boundary, nd = 0.1, 0.3, 0.5, 0.7, and 0.9) by explicitly excluding the surfaces of gray-white matter and gray-pial surface boundaries. A gray-white matter boundary-based registration method implemented in FreeSurfer was used to form the rigid transformation between the functional and the anatomical data. Using this registration file, individuals’ functional time-series volumes were projected to their own five intermediate cortical surfaces by the nearest-neighbor interpolation method. Between-subject averaging was done by morphing individual data through a spherical surface-based coordinate system^60^. The results of the cortical surface reconstruction as well as the functional-anatomical registration were all visually inspected. The registrations for two out of twenty participants were further manually corrected.

### Data analysis

Functional data were corrected for slice timing, motion, and field map-based distortion using the SPM12 software package (http://www.fil.ion.ucl.ac.uk/spm/) and temporally de-trended using a second-order polynomial. After being registered to the surface-based coordinate system, data were smoothed along the surface using a 2D Gaussian kernel at 5 mm full-width at half-maximum (FWHM). To characterize the tonotopy, spectral tuning, and their variabilities across cortical depths, we used a phase-encoded fMRI method published previously^61,62^. Following the procedure, we applied Fourier transform to the fMRI time series at each cortical location from each participant. The phase (*φ*) and the amplitude (*a*) at the presentation frequency (*fp*, 0.033 Hz) were used to construct a sinusoidal activation model.

1 $${model}=a(fp)\times \,\cos (2\pi fp+\phi (fp))$$

The Pearson’s correlation coefficient between the model and the fMRI time series was Fisher-transformed to the standard z-score. All further analyses for individual subjects were limited to cortical locations that responded significantly to the auditory stimulus (z > 1.65; *p* < 0.05). The phase calculated from the rising chirp (${\rm{rc}}$) and falling chirp (fc) data were averaged by the following equation to cancel the hemodynamic response delay. The averaged phase was then linearly transformed to the response latency, which denoted the continuous frequency preference.

2 $${\phi }_{avg}=({\phi }_{rc}+2\pi -{\phi }_{fc})/2$$

To estimate the spectral tuning width, time-series data were segmented and averaged into one chirp presentation block. Ideally, the brain location with preferred frequency *f* 0 may be partially activated by frequencies around *f* 0 with smaller amplitudes. Thus, the averaged time series was fitted by a Gaussian model and the FWHM of the fitted Gaussian curve was calculated. The FWHM expressed in octaves was determined as the spectral tuning width. Hence the low and high values of tuning width correspond to narrow and broad spectral tunings, respectively. For group analysis, the correlation z-scores, frequency preferences, and tuning widths were averaged between participants. To control for multiple comparisons, the functional ROI was determined as cortical locations whose average correlation z-scores exceed a voxel-wise statistic threshold of *p* < 0.01 corrected using false discovery rate (FDR) correction according to the number of voxels in the auditory-related anatomical ROI. The anatomical auditory-related ROI includes Heschl’s gyrus, Heschl’s sulcus, planum temporale, planum polare, and superior temporal gyrus (Destrieux atlas/aparc.a2005s implemented in FreeSurfer). Group results were calculated and displayed only in the mask formed by the intersection between the functional and anatomical ROIs. The resultant auditory cortex ROI did not extend to the most medial portion of the Heschl’s gyrus, probably due to the sharp reduction of the sensitivity at distance far away from the surface coil array. We included this area in the auditory cortex ROI considering the fact that it is a part of the primary auditory core^63,64^. The inter-subject variability was calculated by taking the standard deviation between participants. To calculate the intra-subject variability, we first arbitrarily separated fMRI time series into two data groups with 15 rising chirp blocks and 15 falling chirp blocks, and estimated frequency preferences and tuning widths separately in two groups. After repeating the process 20 times, the intra-subject variability was calculated by taking the standard deviation across 40 (2 groups × 20 times) results for each participant and then averaging across participants. For comparisons of the frequency preference, tuning width, inter- and intra-subject variabilities across five cortical depths, we applied one-way analysis of variance (ANOVA) for repeated measures. The post-hoc *t*-tests were corrected using FDR correction according to the number of cortical depth comparison pairs in our analysis (nd–nd: 0.1–0.3, 0.1–0.5, 0.1–0.7, 0.1–0.9, 0.3–0.9, 0.5–0.9, and 0.7–0.9).

For the intrinsic functional connectivity (iFC) calculation, there was no spatial smoothing during the pre-processing procedure. After regressing out the activation model from the fMRI time series, iFC was computed as the Pearson’s correlation of the residual fMRI signal of each cortical location within core or noncore region of the auditory cortex at each cortical depth. Based on previous studies showing a narrower tuning width at the human primary auditory core^7,8^, we defined the core region as a spatially continuous and narrowly tuned area (with a tuning width threshold of 1.7 octaves) at the Heschl’s gyrus in the average tuning width map across participants and cortical depths. The resultant core region delineation was generally in agreement with previous studies^8,63,64^. The noncore region was defined as rest of the cortical locations in the auditory cortex ROI. To obtain the iFC as a function of frequency preference difference (Δ frequency, log-scale), iFC data corresponding to the Δ frequency between the seed and target vertices (within the same cortical depth) were calculated. We calculated the data with Δ frequency less than three octaves because there were fewer data points at larger Δ frequency. For the group analysis, the individual data were binned with Δ frequency bin edges of 0, 0.1875, 0.375, 0.75, 1.5, and 3 octaves, and then averaged across participants. During all averaging procedures, correlation coefficients were first transformed to Fisher’s z-score, and then averaged and transformed back to correlation coefficients to ensure linearity. To compare the selectivity of feature-dependent iFC between different regions and cortical depths, we quantified the selectivity as the time constant of an exponential decay model (y = R_0_ * exp^−λ * ×^) fit to the Δ frequency-iFC data. Potential inflation of the type-I error due to multiple comparisons of λ profile between regions across five cortical depths were strictly corrected using Bonferroni correction. The iFC as a function of tuning width difference (Δ tuning width, log-scale) was computed using the same method. All calculations were done using MATLAB.

## Data Availability

The authors declare that all the data in this manuscript are available.
